# Efficacy of continuous haemodiafiltration using a polymethylmethacrylate membrane haemofilter in the treatment of sepsis and acute respiratory distress syndrome

**DOI:** 10.1186/cc10985

**Published:** 2012-03-20

**Authors:** M Sakai

**Affiliations:** 1 Shintakeo Hospital, Takeo, Japan

## Introduction

CHDF using a polymethylmethacrylate membrane is currently widely applied for nonrenal indications in Japan; this technique is used in the treatment not only of patients with sepsis but also of those with cytokine-induced critical illness such as acute respiratory distress syndrome (ARDS) and pancreatitis. This study aimed to investigate the clinical efficacy of continuous haemodiafiltration using a polymethylmethacrylate membrane haemofilter (PMMA-CHDF) in the treatment of patients with sepsis and ARDS.

## Methods

Thirty-five patients diagnosed with sepsis (ARDS (*n *= 10), pyelonephritis (*n *= 5), cholangitis (*n *= 5), tsutsugami in Scrub typhus disease (*n *= 1), mamushi snake bite (*n *= 1), haemophagocytic syndrome (*n *= 1), antineutrophil cytoplasmic antibody lung disease (*n *= 1), beriberi heart disease (*n *= 1) and unknown causes (*n *= 8)) were enrolled in this study between August 2010 and November 2011. The common cause for ARDS in older patients was aspiration pneumonia. Our study group comprised 15 men and 20 women, aged 35 to 85 years (median age 68 years).

## Results

Before initiating treatment with the PMMA-CHDF, the average APACHE score of these patients was 17.5 ± 3.6, whereas the average Sepsis-related Organ Failure Assessment score was 6.5 ± 1.3. The duration of PMMA-CHDF treatment was 5.2 ± 2.3 days. Following initiation of PMMA-CHDF treatment, early improvement of haemodynamics was observed, along with an increase in the urine output. The average survival rates of patients were 75.6%. The lowest survival rate among diseases (35%) belonged to the unknown group. The highest survival rate for patients with ARDS was 95%. Moreover, the urine output significantly increased in the survival group.

## Conclusion

The present study suggests that cytokine-oriented critical care using PMMA-CHDF might be effective in the treatment of sepsis and ARDS, particularly in the treatment of ARDS associated with aspiration pneumonia in older patients.

